# Quality appraisal of antibiotic consumption in the community, European Union/European Economic Area, 2009 and 2017

**DOI:** 10.1093/jac/dkab178

**Published:** 2021-08-01

**Authors:** Niels Adriaenssens, Robin Bruyndonckx, Ann Versporten, Niel Hens, Dominique L Monnet, Geert Molenberghs, Herman Goossens, Klaus Weist, Samuel Coenen, the ESAC-Net study group, Reinhild Strauss, Boudewijn Catry, Stefana Sabtcheva, Arjana Tambić Andrašević, Isavella Kyriakidou, Jiří Vlček, Ute Wolff Sönksen, Elviira Linask, Emmi Sarvikivi, Philippe Cavalié, Birgitta Schweickert, Flora Kontopidou, Ria Benkő, Gudrun Aspelund, Karen Burns, Filomena Fortinguerra, Elīna Dimiņa, Rolanda Valintėlienė, Marcel Bruch, Peter Zarb, Stephanie Natsch, Hege Salvesen Blix, Anna Olczak-Pieńkowska, Ana Silva, Gabriel Adrian Popescu, Tomáš Tesař, Milan Čižman, Antonio López Navas, Vendela Bergfeldt, Berit Müller-Pebody

**Affiliations:** 1 Laboratory of Medical Microbiology, Vaccine & Infectious Disease Institute (VAXINFECTIO), University of Antwerp, Antwerp, Belgium; 2 Centre for General Practice, Department of Family Medicine & Population Health (FAMPOP), University of Antwerp, Antwerp, Belgium; 3 Interuniversity Institute for Biostatistics and statistical Bioinformatics (I-BIOSTAT), Data Science Institute, Hasselt University, Hasselt, Belgium; 4 Centre for Health Economic Research and Modelling Infectious Diseases, Vaccine & Infectious Disease Institute (VAXINFECTIO), University of Antwerp, Antwerp, Belgium; 5 Disease Programmes Unit, European Centre for Disease Prevention and Control, Stockholm, Sweden; 6 Interuniversity Institute for Biostatistics and statistical Bioinformatics (I-BIOSTAT), Catholic University of Leuven, Leuven, Belgium

## Abstract

**Objectives:**

The quality of antibiotic consumption in the community can be assessed using 12 drug-specific quality indicators (DSQIs) developed by the European Surveillance of Antimicrobial Consumption (ESAC) project. We compared quality in 2009 and 2017 in the EU/European Economic Area (EEA) and evaluated the impact of using different DDD values (ATC/DDD indices 2011 and 2019) for the 2009 quality assessment using these DSQIs and a joint scientific opinion (JSO) indicator.

**Methods:**

We calculated the 12 DSQIs and the JSO indicator for 2017 and for 2009 for EU/EEA countries able to deliver values. For each of the indicators we grouped the 2017 and 2009 indicator values into four quartiles. To evaluate changes in quality between 2009 and 2017, we used the quartile distribution of the 2009 indicator values in 30 EU/EEA countries as benchmarks. In addition, we compared the quality assessment for 2009 using the ATC/DDD indices 2011 and 2019.

**Results:**

In 2017, a difference in the quality of antibiotic consumption in the community between northern and southern EU/EEA countries remained, but also several eastern EU/EEA countries shifted towards lower quality. Quality of antibiotic consumption decreased between 2009 and 2017 in particular indicator values for penicillin, quinolone, relative β-lactam and broad- versus narrow-spectrum antibiotic consumption, and seasonal variation. Using different ATC/DDD indices did not substantially change countries’ ranking based on their DSQI values.

**Conclusions:**

The quality of antibiotic consumption in the community as measured by the DSQIs further decreased between 2009 and 2017, especially in Southern and Eastern European countries. A continuous effort to improve antibiotic consumption is essential to reduce antibiotic consumption in general and the use of broad-spectrum antibiotics in particular.

## Introduction

Quality assessment and improvement in healthcare is a major issue in many countries.^1^^,^^2^ Information on quality of healthcare is being demanded by policy makers, healthcare professionals and the general public.^3^ Prescribing of medicines also has a major influence on well-being and accounts for a substantial part of healthcare expenditure.^4^ If we want to improve the consumption of antibiotics, we have to be able to measure the quality of antibiotic consumption in Europe. Benchmarking by comparisons between countries has proved to be an important stimulus to quality improvement, in general, but also for antibiotic consumption in particular.^5^ In 2007, the European Surveillance of Antimicrobial Consumption (ESAC) project published a set of 12 drug-specific quality indicators (DSQIs) for antibiotic consumption in the community (i.e. primary care sector) and calculated the indicator values for 2004.^6^ It was concluded that these indicators could be used to describe antibiotic consumption in the community and to assess the quality of national antibiotic prescribing patterns in Europe. In 2011, a quality appraisal of antibiotic consumption in Europe was published to evaluate the quality of antibiotic consumption in the community in 2009 and to evaluate changes in quality between 2004 and 2009.^7^

In 2019, the DDDs of several substances were updated by WHO.^8^^,^^9^ In particular, the DDD for amoxicillin (J01CA04) and amoxicillin/clavulanic acid (J01CR02) changed for oral administration from 1 g to 1.5 g to better approximate the dose consumed in daily practice.^8^^,^^9^ Retrospectively, this change could be applied to previous quality assessments and would cause different values for several indicators. Therefore, we chose to update the quality assessment for 2009 and assess the impact of using the ATC/DDD index 2019.^10^

In addition, on request of the European Commission, ECDC, the European Food Safety Authority (EFSA) and EMA published a Joint Scientiﬁc Opinion (JSO) on a list of outcome indicators with regard to surveillance of antimicrobial resistance and antimicrobial consumption in humans and food-producing animals to monitor the EU/European Economic Area (EEA) country activities on prudent use of antimicrobials.^11^ In parallel with the respective ESAC quality indicator, the JSO proposes an indicator using a modified ratio of consumption of broad-spectrum antibiotics {J01[CR+DC+DD+(F-FA01)+MA]} to the consumption of narrow-spectrum antibiotics [J01(CA+CE+CF+DB+FA01)]. In this article, we will also present results for this ECDC/EFSA/EMA JSO indicator. However, the primary objective of this article, which is one of a series of articles,^9^^,^^12–19^ is to present a detailed quality assessment of antibiotic consumption in the community in EU/EEA countries in 2017 and an assessment of changes in quality of this consumption between 2009 and 2017.

## Methods

The methods for collecting data on consumption of systemic antibiotics are described in the introductory article of this series.^9^ Antibiotic consumption in the community was expressed in DDD per 1000 inhabitants per day using the ATC/DDD index 2019.^10^ The quality of antibiotic consumption in the community in 2017 was assessed for each country by calculating the indicator values for each of the 12 DSQIs defined by the ESAC project and for the ECDC/EFSA/EMA JSO indicator (Table 1) using data on antibiotic consumption in 2017 available from the European Surveillance of Antimicrobial Consumption Network (ESAC-Net) database and grouping these values into quartiles.^6^^,^^11^ Next, for the same indicators, the 2009 indicator values were grouped into four quartiles according to the quartile distribution of the 2009 indicator values using the 2019 DDD values.

**Table 1.** Quality indicators for outpatient antibiotic consumption in the community

No.	Label	Description
1	J01	Consumption of antibacterials for systemic use (J01) expressed in DDD per 1000 inhabitants per day
2	J01C	Consumption of penicillins (J01C) expressed in DDD per 1000 inhabitants per day
3	J01D	Consumption of cephalosporins (J01D) expressed in DDD per 1000 inhabitants per day
4	J01F	Consumption of macrolides, lincosamides and streptogramins (J01F) expressed in DDD per 1000 inhabitants per day
5	J01M	Consumption of quinolones (J01M) expressed in DDD per 1000 inhabitants per day
6	J01CE%	Consumption of β-lactamase-sensitive penicillins (J01CE) expressed as percentage^a^
7	J01CR%	Consumption of combination of penicillins, including β-lactamase inhibitor (J01CR) expressed as percentage^a^
8	J01DD+DE%	Consumption of third- and fourth-generation cephalosporins [J01(DD+DE)] expressed as percentage^a^
9	J01MA%	Consumption of fluoroquinolones (J01MA) expressed as percentage^a^
10	J01_B/N	Ratio of the consumption of broad-spectrum antibiotics {J01[CR+DC+DD+(F-FA01)]} to the consumption of narrow-spectrum antibiotics [J01(CE+DB+FA01)]
11	J01_SV	Seasonal variation of the total antibiotic consumption (J01)^b^
12	J01M_SV	Seasonal variation of quinolone consumption (J01M)^b^
13	J01_B/N_JSO	Ratio of the consumption of broad-spectrum antibiotics {J01[CR+DC+DD+(F-FA01)+MA]} over narrow-spectrum antibiotics [J01(CA+CE+CF+DB+FA01)]

a Percentage of the total consumption of antibacterials for systemic use (J01) expressed in DDD per 1000 inhabitants per day.

b Overconsumption in the winter quarters (October–December and January–March) compared with the summer quarters (July–September and April–June) of a 1 year period starting in July and ending the next calendar year in June, expressed as percentage: [DDD (winter quarters)/DDD (summer quarters)−1]×100.

Indicator values within the first quartile [i.e. values ≤percentile 25 (p25)] suggest better quality than indicator values within the second quartile (i.e. p25 <values ≤p50), which suggest better quality than indicator values within the third quartile (i.e. p50 <values ≤p75), which suggest better quality than indicator values within the fourth quartile (i.e. values >p75) for that indicator.^6^ Countries were ranked firstly according to the number of indicator values within the fourth quartile, secondly according to the number of indicator values within the third quartile, and thirdly according to the number of indicator values within the second quartile, taking into account the total number of available indicator values. The ECDC/EFSA/EMA JSO indicator was not included in this ranking.

We compared the quality assessment for 2009 using the ATC/DDD indices 2011 and 2019.^10^^,^^20^ To evaluate changes in quality between 2009 and 2017, we used the quartile distribution of the 2009 indicator values as benchmarks for the 2017 values. Only countries able to deliver data for both years were included in this comparison.

## Results

**Figure 1.**
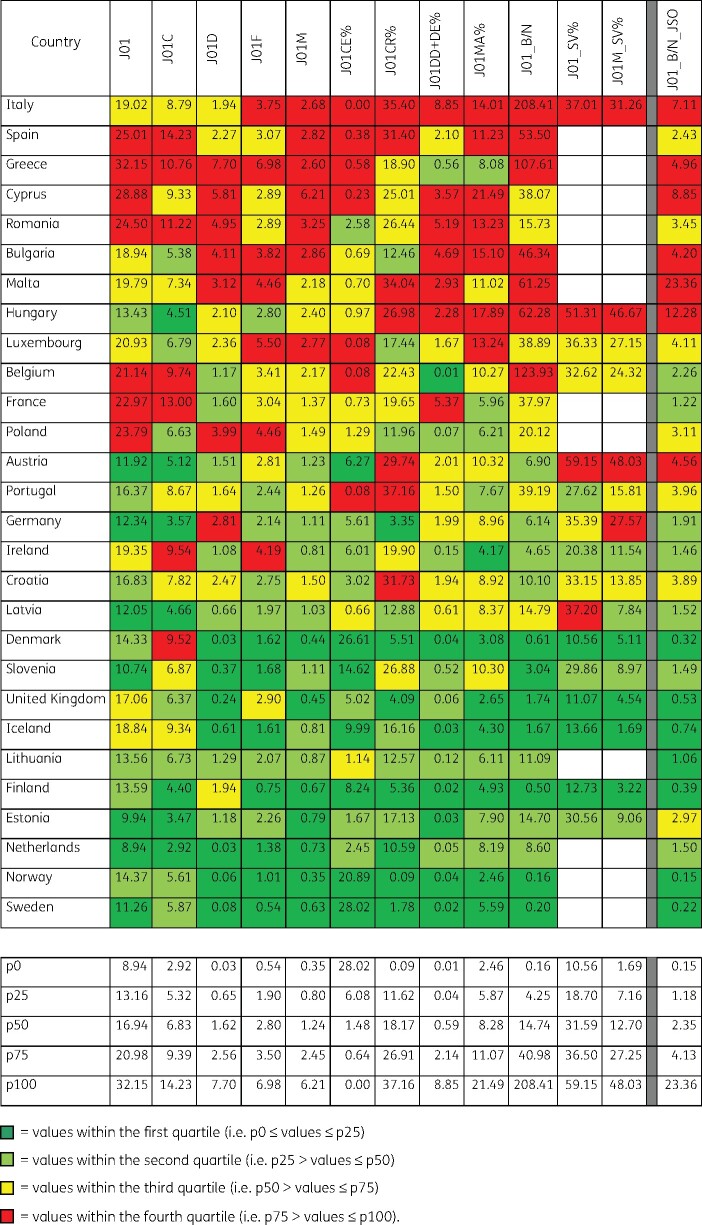
Quality indicators for outpatient antibiotic consumption in the community, 2017 (ATC/DDD index 2019), 28 EU/EEA countries grouped into four quartiles based on 2017 quartile distribution. For Cyprus and Romania, total care, i.e. community + hospital sector, data were used.

Figure 1 shows the 2017 indicator values for the 28 EU/EEA countries that reported 2017 antibiotic consumption data (i.e. all EU/EEA countries except Czechia and Slovakia), grouped into four quartiles and ranked according to quality. Based on this ranking, several Northern European countries (Finland, Iceland, Lithuania, the Netherlands, Norway, Sweden and the United Kingdom) showed a better quality of antibiotic consumption in the community than Southern European countries [Cyprus (total care data), Greece, Italy, Malta and Spain] and several Eastern European countries [Bulgaria, Hungary and Romania (total care data)]. Compared with the previous quality assessment in 2011, several Eastern European countries ranked lower in quality in 2017 compared with 2009.

**Figure 2.**
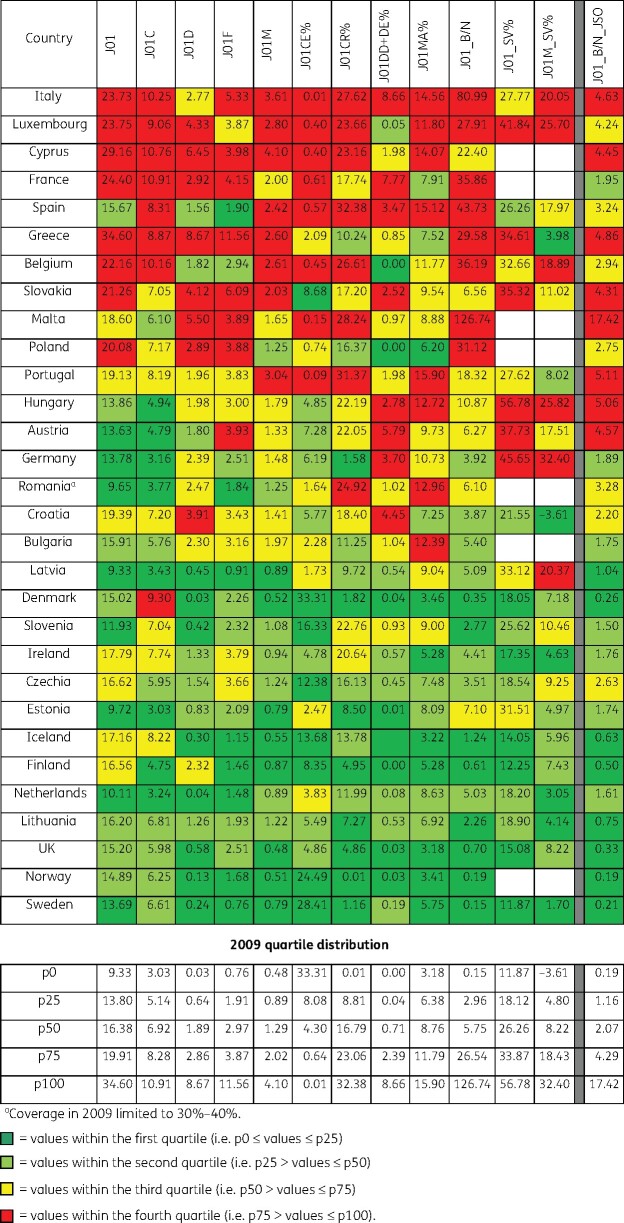
Quality indicators for outpatient antibiotic consumption in the community, 2009 (ATC/DDD index 2019), 30 EU/EEA countries grouped into four quartiles based on 2009 quartile distribution. For Cyprus, Lithuania and Romania, total care, i.e. community plus hospital sector, data were used.

Figure 2 shows the 2009 indicator values for the 30 EU/EEA countries that reported 2009 antibiotic consumption data, grouped into quartiles, and ranked according to quality. By using the ATC/DDD index 2019 instead of the ATC/DDD index 2011,^20^ Lithuania ranked substantially higher in quality (i.e. their ranking changed by four or more positions) than other countries, while for Denmark the opposite was observed.

**Figure 3.**
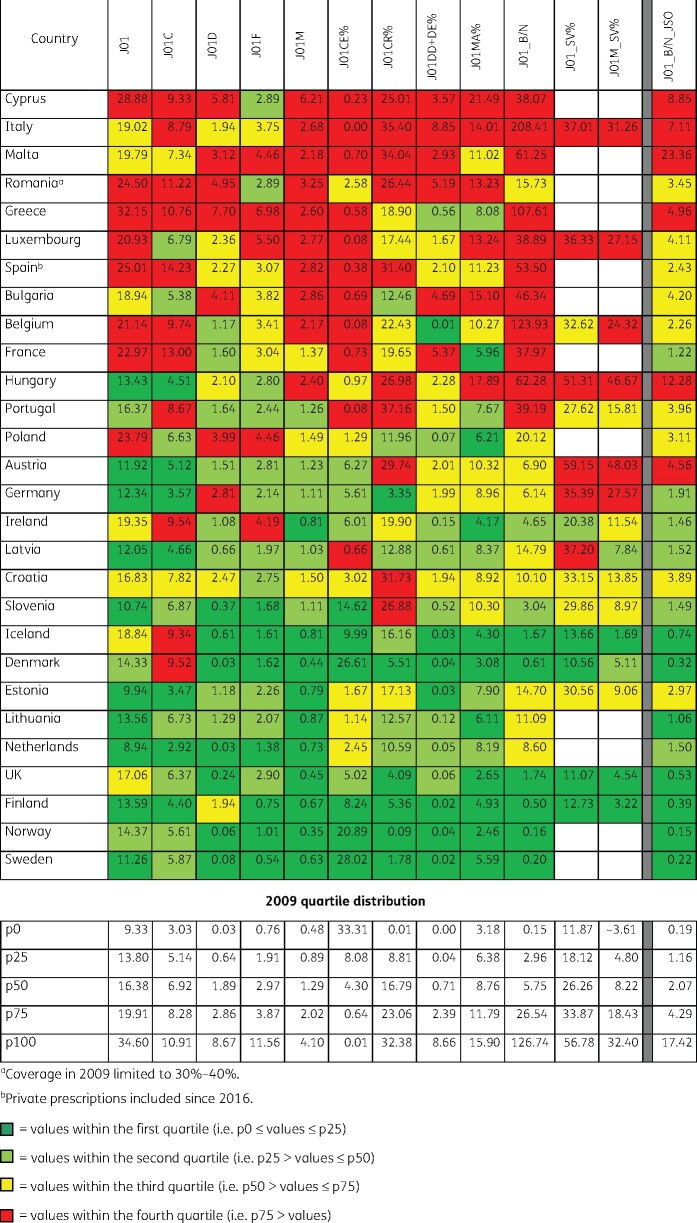
Quality indicators for outpatient antibiotic consumption in the community, 2017 (ATC/DDD index 2019), 28 EU/EEA countries grouped into four quartiles based on 2009 quartile distribution. For Cyprus and Romania, total care, i.e. community plus hospital sector, data were used.

As shown in Figure 3, quality of antibiotic consumption in the community declined between 2009 and 2017. There were 19 more indicator values within the fourth quartile and six more indicator values within the first quartile in 2017 compared with 2009; this was at the expense of 16 fewer indicator values within the second quartile and 9 fewer indicator values within the third quartile.

The most substantial shifts towards lower quality were observed for the following quality indicators: penicillin consumption in DDD per 1000 inhabitants per day (J01C_DID), quinolone consumption in DDD per 1000 inhabitants per day (J01M_DID), the proportional consumption of β-lactamase-sensitive penicillins (J01CE_%), the proportional consumption of combinations of penicillins, including β-lactamase inhibitors (J01CR_%), the ratio of broad- to narrow-spectrum antibiotics (J01_B/N), the seasonal variation of total antibiotic consumption (J01_SV) and the seasonal variation of quinolone consumption (J01M_SV%).

For three quality indicators, i.e. consumption of antibacterials for systemic use in DDD per 1000 inhabitants per day (J01_DID), quinolone consumption in DDD per 1000 inhabitants per day (J01M_DID) and consumption of fluoroquinolones (J01MA) expressed as percentage (J01MA_%), shifts towards better quality were observed.

The quality indicator J01M_DID showed the highest degree of polarization (20 indicator values in the first and last quartile versus 8 indicator values in the second and third quartiles).

## Discussion

In 2017, there still was an important North–South divide when considering the quality of antibiotic consumption. Several Eastern European countries [Bulgaria, Hungary and Romania (total care data; coverage in 2009 limited to 30%–40%)] ranked lower in quality in 2017 than 2009.

The interpretation of the 2017 values using the 2009 quartile distribution suggests that total antibiotic consumption (J01_DID) further improved (10 of the 28 values in the first quartile), which was caused by a shift of some countries from the second quartile to the first quartile. There was no shift from the countries in lower quality quartiles (third and fourth quartile). This suggests that the countries with higher quality further improved between 2009 and 2017 whereas the countries with lower quality remained low in quality. This was also visible in the indicator considering quinolone consumption in DDD per 1000 inhabitants per day (J01M_DID). Countries with better quality in 2009 seemed to reduce their quinolone consumption whereas countries with lower quality in 2009 seemed to increase their quinolone consumption or did not substantially reduce it.

The WHO continuously reviews its ATC/DDD methodology so the DDDs better approximate the daily doses consumed in daily practice. Changes in DDDs are to be kept at a minimum and avoided as far as possible, as too many changes are disadvantageous for long-term studies on drug utilization.^20^ There have not been many changes in the DDDs for antibiotics, but the 2019 version of the ATC/DDD index introduced large and important changes for several substances. Most importantly, the DDD for amoxicillin (J01CA04) and amoxicillin/clavulanic acid (J01CR02) changed from 1 g to 1.5 g for oral use.^8^ In contrast to the results of the study by Charra *et al.*,^21^ this change did not dramatically influence ranking or alter conclusions on the quality of antibiotic consumption compared with the assessment with previous DDD values (ATC/DDD index 2011). The largest shifts in ranking were observed for Lithuania (higher quality) and Denmark (lower quality). However, interpretation of ranking should be done with caution because these indicators are not independent. Therefore, we strongly advise that each country critically appraises its quality indicator values. In particular, country rankings are influenced by shifts in total antibiotic consumption and in penicillin consumption in DDD per 1000 inhabitants per day. For example, in Denmark, consumption of extended-spectrum penicillins and combinations with a β-lactamase inhibitor (amoxicillin and amoxicillin/clavulanic acid) is limited compared with the other EU/EEA countries, which is not necessarily a sign of lower quality of antibiotic consumption in the country. Countries with high consumption of extended-spectrum penicillins and combinations with a β-lactamase inhibitor benefit the most from the 2019 alteration in DDDs, but the proportional consumption of these two substances could also be considered as a tool for quality assessment.^22^

For this reason, the ECDC, EFSA and EMA JSO proposed an indicator using a modified ratio of consumption of combinations of penicillins (J01CR; including the combination of amoxicillin/clavulanic acid), broad-spectrum cephalosporins (J01DC and J01DD), broad-spectrum macrolides (J01F, excluding J01FA01) and ﬂuoroquinolones (J01M) to the consumption of narrow-spectrum penicillins (J01CE), extended-spectrum penicillins (J01CA, including amoxicillin), penicillinase-resistant penicillins (J01CF), narrow-spectrum cephalosporins (J01DB) and narrow-spectrum macrolides (J01FA01). Based on this indicator, Denmark ranked in the first quartile. Although the indicator was not validated by a consensus procedure, we invite countries to also consider their position for this indicator. For example Estonia, which has all indicator values within the first and second quartile when considering the 12 ESAC DSQIs, ranked in the third quartile considering the ECDC/EFSA/EMA JSO indicator. The authors already noted that this indicator predominantly reﬂects antimicrobial consumption in the community and should not stand alone, but be used in combination with other indicators, e.g. hospital antimicrobial consumption indicators.^11^

In addition to this, we again emphasize the need for consumption data that are related to clinical information to assess the quality of antibiotic consumption.^7^ The ESAC project had also developed disease-specific quality indicators for antibiotic prescribing in the community, i.e. antibiotic prescribing rates, prescribing of recommended antibiotics and prescribing of quinolones for seven main indications.^23^ National and even regional quality assessments using these tools will help countries and local practices to better understand prescribing habits and identify opportunities for improvement.^24–34^ For example in Belgium, such a quality assessment identified the unavailability of narrow-spectrum penicillins (J01CE) in the country due to industry stock cuts,^24^ which calls for measures to make these antibiotic substances accessible again.^35^

In conclusion, increased consumption of broad-spectrum antibiotics in DDD per 1000 inhabitants per day and an increasing seasonal variation were observed in EU/EEA countries between 2009 and 2017. The quality of antibiotic consumption further decreased between 2009 and 2017, especially in Southern and Eastern European countries. Therefore, sustained efforts to improve antibiotic consumption in the community are essential to reduce antibiotic consumption in general and the consumption of broad-spectrum antibiotics in particular. The 2017 values of the ESAC DSQIs for antibiotic consumption in the community and the ECDC/EFSA/EMA JSO indicator allow individual countries to assess their position in relation to other countries, and we hope this will trigger actions to improve antibiotic consumption.
